# The dengue situation in Africa

**DOI:** 10.1179/2046904712Z.00000000048

**Published:** 2012-05

**Authors:** Fred Were

**Affiliations:** Department of Paediatrics, University of Nairobi, Kenya

**Keywords:** Dengue, Africa, Epidemic, Epidemiology, Race

## Abstract

Dengue outbreaks and epidemics have been reported in all regions of Africa, and it is believed that all four dengue virus serotypes are in circulation. Available data suggest that dengue is endemic to 34 African countries and that *Aedes aegypti* mosquitoes – the primary vector for dengue transmission – are known to be present in all but five countries. Whether populations in Africa are susceptible to dengue at the same rates as in Asia and Latin America is difficult to determine from the available data. Several factors may affect the transmission of dengue in Africa, including vector efficiency, viral infectivity, host vulnerability and environmental factors, such as increasing urbanisation. Current dengue prevention strategies in Africa focus on vector control, although the primary aim of such efforts is typically the prevention of malaria. Further research is needed to characterise the epidemiology of dengue in Africa and to better understand the factors involved in differences in vulnerability to dengue across Africa.

## Dengue Risk Areas and Epidemic Activity in Africa

**Figure 1:**
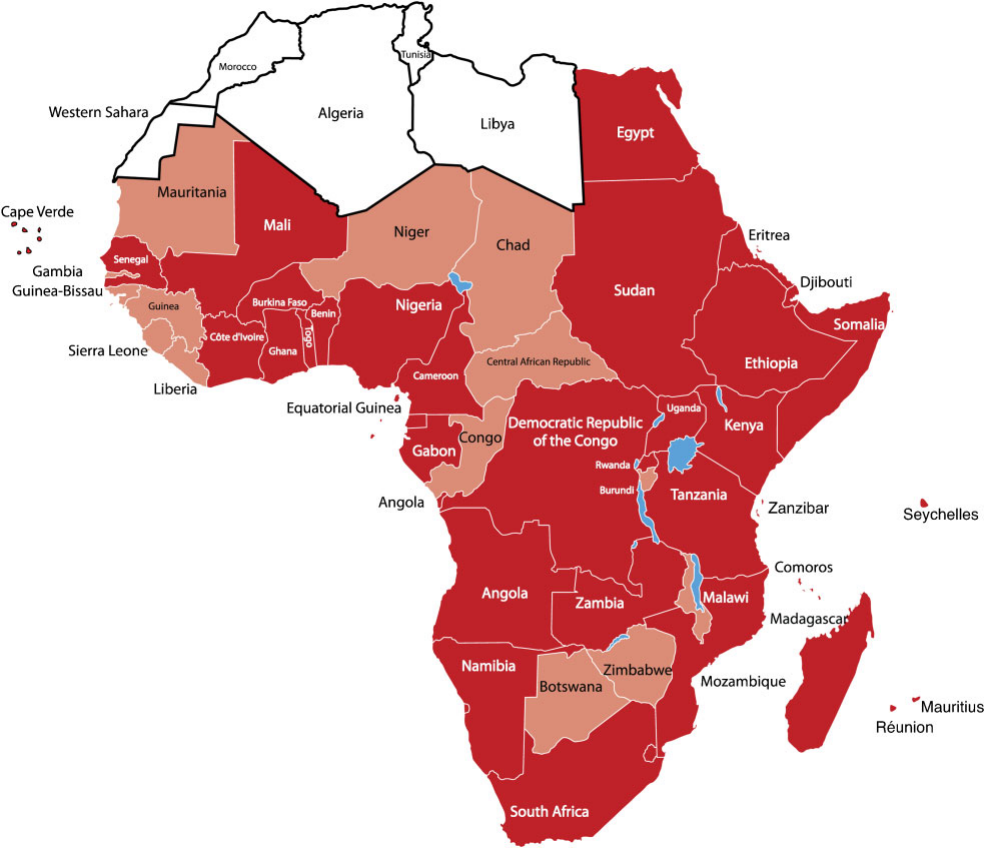
Dengue and *Aedes aegypti* in Africa.^2^ The 34 countries in a dark colour indicate those in which dengue has been reported, including dengue reported only in travellers, and the presence of *Aedes aegypti* mosquitoes. Light-coloured areas indicate the 13 countries in which dengue has not been reported but where *Aedes aegypti* mosquitoes are present. White indicates five countries for which data are not available.

The World Health Organization (WHO) currently estimates that there are 50 million cases of dengue infection each year, with approximately 500,000 requiring hospitalisation. Of these severe dengue cases, approximately 5% will die.^1^ *Aedes aegypti* mosquitoes – the primary vector for dengue transmission – are known to be present in all but five countries (Western Sahara, Morocco, Algeria, Tunisia and Libya), for which data are not available (Fig. 1).^2^

Dengue epidemics have been reported in Africa since the 19th century, in countries including Zanzibar (1823, 1870), Burkina Faso (1925), Egypt (1887, 1927), South Africa (1926–1927), and Senegal (1927–1928).^2^ Between 1960 and 2010, 20 laboratory-confirmed outbreaks were reported in 15 African countries, with most occurring in Eastern Africa. All four dengue virus (DENV) serotypes have been isolated in Africa, with DENV2 reported to cause the most epidemics.^2^

Available data suggest that dengue is endemic to 34 countries across all regions of Africa (Table 1, Fig. 1).^2^ Of these, 22 have reported local transmission, which is laboratory-confirmed in 20 countries, while two (Egypt and Zanzibar) do not have laboratory confirmation. The remaining 12 countries have only diagnosed dengue in travellers who had returned to non-endemic regions.

**Table 1:** Countries reporting transmission since 2000^2^

	Countries reporting overall transmission since 2000	Countries reporting transmission to travellers since 2000
West Africa	Burkina Faso	Equatorial Guinea
	Ghana	Gabon
	Senegal	Mali
	Cameroon	Togo
	Côte d’Ivoire	
	Cape Verde	
	Equatorial Guinea	
	Gabon	
	Mali	
	Togo	
East Africa	Madagascar	Ethiopia
	Zimbabwe	Tanzania
	Ethiopia	Uganda
	Tanzania	
	Uganda	
Central Africa	Angola	Angola
	Democratic Republic of the Congo	Democratic Republic of the Congo
Southern Africa	Namibia	Namibia
	Zambia	Zambia

More detailed epidemiological data are required to assess the impact of dengue in Africa. Data on incidence and prevalence are not available for Africa, despite the fact that outbreaks have been recorded.^2^ Under-reporting and under-recognition of dengue are key concerns, since the majority of febrile illnesses are treated as presumptive malaria.^2^

## Factors Influencing Transmission of Dengue Virus

### Vector efficiency

The principal vector for dengue fever, *A. aegypti*, originated in Africa and has spread throughout the continent and to other tropical regions.^2^ Other *Aedes* species present in Africa, which also act as potential vectors, include *A. albopictus*, *A. africanus* and *A. luteocephalus*.

Susceptibility of different mosquito strains to DENV has been shown to vary geographically. African strains of *A. aegypti* and *A. albopictus* have shown uniformly lower susceptibility to all four subtypes of DENV in laboratory settings.^3^–^5^ This reduced vector efficiency for dengue transmission may explain the apparent lower than expected prevalence in Africa, though further study is urgently needed.^2^

### Viral infectivity

Dengue is caused by four genetically related but antigenically different viruses (DENV1–4) and all four serotypes are present in Africa and maintained in enzootic cycles, most likely between non-human primates and arboreal mosquitoes.6,^7^

Although the enzootic forms of DENV may be becoming less infective in Africa, there is still a potential for endemic forms of the virus to emerge from sylvatic cycles between mosquitoes and non-human primates.^7^ However, more infective varieties from Asia and the Western Pacific may be increasing in Africa due to travel, and it is thought that recent African outbreaks are due to spill-over from other regions rather than from sylvatic cycles.2,^8^

### Host vulnerability

Race may be a factor in resistance to dengue infection, with some studies suggesting that black patients are more resistant. During a 1981 epidemic in Cuba, a country with a majority white but significant black population, white individuals were disproportionately susceptible to dengue infection, as well as to severe dengue and fatality.9,^10^ Dengue cases were reported in Los Angeles in 1998, but only among Hispanic and white ethnic groups.^1^ Genetic polymorphism in cytokines and coagulation proteins has been proposed as a potential mechanism conferring resistance to black individuals.^1^

Age is also a key factor in terms of vulnerability to dengue infection. While dengue fever is often thought of as a childhood disease, it has been observed that the incidence of dengue haemorrhagic fever is increasing in older age groups.^1^

Travellers may be more susceptible to dengue infection than locals. This is particularly the case for travellers from non-endemic to endemic areas. It is not certain whether partial immunity among the locals may be responsible for this phenomenon.^11^

### Environmental factors

Increasing urbanisation creates favourable conditions for increased transmission, increases in the vector population and perhaps changes in the ecological balance of different strains.^12^ Since the 1950s, there has been a three-fold increase in urban population density across Africa.^13^ Informal settlements can be associated with increased risk of dengue infection, since artificial water collection increases the available habitat for vectors.

Recent reports are however showing a global increase in rural epidemics, especially in Africa. This is one of the emerging paradigms of dengue fever. It is not certain whether this is related to modernisation of villages or deforestation shifting the vector nearer to settlements.^1^

## Recent Epidemics and Dengue Prevention in Africa

Dengue epidemics have occurred in all regions of Africa in the 5 years between 2006 and 2011 (Table 2).^2^ It is likely that all four subtypes of the dengue virus are present but the lack of formal laboratory testing or surveillance initiatives means that it is difficult to verify this.

**Table 2:** Overview of most recent epidemics in Africa^2^

Region	Year	Serotype	Reach
West Africa	2008–2009	3	Local and exported
East Africa	2009–2010	Unknown, 2	Local
Central Africa	2006–2007	Unknown	Local and exported
Southern Africa	2006	Unknown	Exported

Given the occurrence of dengue epidemics and the paucity of diagnostic infrastructure, preventative measures are required across Africa. Current dengue prevention strategies in Africa focus on vector control, although the primary aim of such efforts is typically the prevention of malaria.14,^15^ Reduction of breeding sites and targeted destruction of vector populations with insecticides are used throughout all regions. Insecticide-impregnated bed nets are also provided in many regions, but inconsistent provision and low uptake may attenuate the benefits of this measure.^15^ Personal protection is available for travellers, including insect repellants and information to raise dengue awareness.

Robust surveillance programmes must be established in Africa to accurately determine the true burden of dengue and – particularly in the dengue vaccine era – assess the effectiveness of prevention programmes.

## Conclusions

Dengue fever outbreaks and epidemics are frequently reported in Africa, with recent outbreaks occurring predominantly in the Eastern region.2,^16^ However, many outbreaks in Africa are not well characterised, due to the poor surveillance infrastructure and under-recognition of the disease. Whether populations in Africa are susceptible to dengue at the same rates as in Asia and Latin America is difficult to determine from the available data. The African population is thought to be less vulnerable to infection than other ethnic groups, and there may be differences in terms of vector efficiency and viral infectivity between Africa and other dengue-endemic regions. However, environmental factors, including rapidly rising urbanisation in Africa, are associated with increased transmission. Further research is needed to characterise the epidemiology of dengue in Africa and to understand in more detail the factors involved in differences in vulnerability to dengue across Africa.
